# Open access to sequence: Browsing the *Pichia pastoris *genome

**DOI:** 10.1186/1475-2859-8-53

**Published:** 2009-10-16

**Authors:** Diethard Mattanovich, Nico Callewaert, Pierre Rouzé, Yao-Cheng Lin, Alexandra Graf, Andreas Redl, Petra Tiels, Brigitte Gasser, Kristof De Schutter

**Affiliations:** 1 Department of Biotechnology, University of Natural Resources and Applied Life Sciences, Vienna, Austria; 2 School of Bioengineering, University of Applied Sciences FH-Campus Wien, Vienna, Austria; 3 Unit for Molecular Glycobiology, Department for Molecular Biomedical Research, VIB, Ghent-Zwijnaarde, Belgium; 4 Unit for Molecular Glycobiology, L-ProBE, Department of Biochemistry and Microbiology, Ghent University, Ghent-Zwijnaarde, Belgium; 5 Department of Plant Systems Biology, VIB, Ghent-Zwijnaarde, Belgium; 6 Department of Plant Biotechnology and Genetics, Ghent University, Ghent, Belgium; 7 Department for Biomedical Molecular Biology, Ghent University, Ghent-Zwijnaarde, Belgium

## Abstract

The first genome sequences of the important yeast protein production host *Pichia pastoris *have been released into the public domain this spring. In order to provide the scientific community easy and versatile access to the sequence, two web-sites have been installed as a resource for genomic sequence, gene and protein information for *P. pastoris*: A GBrowse based genome browser was set up at  and a genome portal with gene annotation and browsing functionality at . Both websites are offering information on gene annotation and function, regulation and structure.

In addition, a WiKi based platform allows all users to create additional information on genes, proteins, physiology and other items of *P. pastoris *research, so that the *Pichia *community can benefit from exchange of knowledge, data and materials.

## Commentary

Modern biological research requires genome sequence information of the organisms of interest for numerous applications: the development of transcriptomic methods like DNA microarrays relies on genome data, proteomics needs a genome sequence for efficient identification of proteins, metabolic modelling and flux analysis is based on the knowledge of ideally all enzymatic reactions encoded in the genome of an organism. Systems biology, as the synthesis of the above mentioned techniques [1], relies on comprehensive genome sequence data. Systems biology is most advanced for a few model organisms, for which genome sequencing has been an international challenge funded with public support. Systems biotechnology, the application of these approaches to biotechnological strain and process development, faces the same needs [2]. However, genome sequencing of biotechnologically relevant organisms has mainly been pursued with corporate support, and the results were kept confidential over years for commercial exploitation. A major disadvantage of this strategy is the delay of basic research related to these organisms, negatively affecting the knowledge of organisms with the highest relevance for industry.

One such example is the yeast *Pichia pastoris*, widely used for heterologous protein production (reviewed in [3,4]), but also for the production of metabolites [5,6]. The major research areas towards implementing *P. pastoris *as a production host for heterologous proteins are engineering of glycosylation [7-9] and protein folding and secretion (reviewed in [10]). A draft genome sequence has been available commercially since appr. 5 years and omics methods have been developed based on this sequence (transcriptomics [11,12]; proteomics [13]; metabolic flux analysis ()[14,15]), but the strict obligation to keep sequence information confidential has hampered publication of relevant data and collaborations, so that the community could not benefit from exchange of knowledge, data and materials.

To bridge this gap we have published the genome sequences of two *P. pastoris *strains, DSMZ 70382 [16] and GS115 [17], obtained with next generation sequencing technologies. Versatile access to genome sequences is a prerequisite for efficient utilisation of the information. Therefore a genome browser was set up at [18] with a main focus on *P. pastoris *DSMZ 70382 and a genome portal with the gene annotation and browsing functionality for *P. pastoris *GS115 at [19].

Both of these *Pichia *sites serve as a resource for genomic sequence data and gene and protein information for *P. pastoris*. The genome browser (GBrowse for DSMZ 70382 and AnnoJ [20] for GS115) allows users to view and navigate genomic sequences including non-translated regions of the genome. BLAST searches for comparing any query sequence against the *P. pastoris *dataset, full text searches and gene/sequence resources (Get Sequence) serve to retrieve, display and analyze a gene or sequence in many ways, such as protein translation. In the near future, a comparison of the genome of different strains will be added to both genome browsers.

The genome browser of *P. pastoris *DSMZ 70382 is based on the Generic Genome Browser (GBrowse) which consists of a web interface and a database backend. The system was developed by the Generic Model Organism Database project [21,22] for the purpose of exploring genomic sequences together with annotated data. GBrowse has already been used successfully in various genome database projects like SGD, FlyBase or WormBase and its functionality will therefore be familiar to many researchers. The browser simultaneously provides a bird's eye view and detailed views of the genome and facilitates easy navigation through the genome using its zoom capacity. A flexible display of a variety of features, including genes, proteins, RNAs, GC content and restriction sites, on separated customizable tracks permits the user to adapt the browser to his or her needs. The visualization of Microarray probe locations allow for the direct access to specific probe sequence and location of published microarray designs [12]. The *Pichia *Genome Browser further allows locating DNA or protein sequence patterns, to design sequencing and PCR primers and to display restriction maps for a sequence. Several search functions are implemented, including a full text search of the gene annotation. Each gene has a details page where further information about the gene such as its annotation or assigned Gene Ontology (GO) terms [23] is displayed. Apart from the DNA, the coding and the translated sequence of a gene, an up- or downstream region can be specified to be displayed on this page. At the bottom of each details page, links allow users to directly send the specific sequence to other analysis tools such as BLAST. Furthermore, the results of a precalculated InterProScan pattern search [24] are displayed for each annotated protein and can be accessed through the respective link. A comments section enables researchers to add information to their genes of choice. Data downloads are available either in the format of decorated FASTA files or gff files which include gene annotation. Future work on the genome browser of *P. pastoris *DSMZ 70382 will include a genome snapshot which will summarize the status of annotation and the distribution of gene products among functional groups. Batch download processes and an extension of the tools section are planned as well as a platform for the community to share experiences and knowledge in order to promote collaboration. Tutorials for GBrowse are available at [25] or [26].

Except the basic genome browsing and search function, the genome portal of GS115 strain also provides a comprehensive protein-coding gene annotation by the BOGAS (Bioinformatics Gent Online Genome Annotation System). The BOGAS is a gene centric concept, which means the information is provided based on the information related to the gene. Each gene has it's own annotation page which provides an overview of the gene information including the annotator, gene function, gene ontology, protein domain, protein homologs, gene structure, CDS and protein. The annotator information tells who and when annotated this gene and the history log to go back to previous version. Gene function field is filled by annotators with the full gene function and a dictionary to provide a standardized gene nomenclature (short name). The BOGAS system automatically updates the protein information to provide the gene ontology and protein domain by InterProScan, the protein homologs and the multiple alignment by BLASTP and MUSCLE [27] when the user updates the gene structure.

The most important feature of BOGAS system is that it allows the registered users to update the information. Users can correct existing gene structure or create new genes by the annotation software (Artemis [28] or GenomeView [29]) and contribute their expert biological domain in the gene function field. Since the BOGAS provides the history log function, other experts can update the information and people in the community can trace these changes in few clicks. The full text search function in BOGAS can search across locus id, protein domain, genomic location and annotator information. The BLAST function also provides bidirectional link between the query sequence and the possible gene or genomic region. After running the sequence similarity search to fish out the candidate gene or genomic sequence, the user will be linked between the BLAST search result and the corresponding gene region.

As it has been adopted already to a large extent, we suggest that *P. pastoris *gene names should follow the format established for *S. cerevisiae *gene names. A detailed guide to *S. cerevisiae *nomenclature has been published in Trends in Genetics [30]. The gene name should consist of three letters followed by an Arabic number (e.g. *TPI1*). Where *P. pastoris *and *S. cerevisiae *genes appear to be orthologous, they should share the same gene name. The use of prefixes adds clarity to papers discussing genes from different species that share a name (e.g., Pp*URA3 *vs. Sc*URA3*), but the gene names themselves do not include the prefix.

These two *Pichia pastoris *genome sites have been developed as a service for the scientific community. The remote annotations can be added either by informing the authors or through the BOGAS system. The WiKi based platform will allow to create additional information on genes, proteins, physiology and other items of *P. pastoris *research. We invite the *P. pastoris *community to join our efforts by providing new information on gene annotation, function, regulation and structure.

## Competing interests

The authors declare that they have no competing interests.

## Authors' contributions

All authors contributed equally to this manuscript.
